# Support or Suppress? Research on the Mechanism of Employee’s GNS on Innovation Performance: From the Perspective of Status Competition

**DOI:** 10.3389/fpsyg.2022.895266

**Published:** 2022-06-22

**Authors:** Yuhong Tang, Zhenkuo Ding, Xiwu Hu, Ran Tao

**Affiliations:** ^1^College of Economics and Management, Nanning Normal University, Nanning, China; ^2^School of Economics and Management, Guangxi Normal University, Guilin, China; ^3^School of Economics and Management, Qinghai Minzu University, Xining, China; ^4^Qinghai Key Laboratory of High-value Utilization of Characteristic Economic Plants, Xining, China; ^5^School of Economics and Management, Wuhan University, Wuhan, China

**Keywords:** growth need strength, leader–member exchange, perceived status threat, status competition, innovation performance

## Abstract

The purpose of this paper is to investigate how supervisor’s mental state and behavior choice affect the relationship between employees’ strong growth need (GNS) and their innovation performance. Using 210 sets of supervisor-subordinate dyads data from two-wave survey, this research reveals that GNS has a significant positive effect on innovation performance, and leader–member exchange (LMX) mediates the effect of GNS on innovation performance. Supervisor perceived status threat moderates the relationship between GNS and LMX, such that this relationship gets weaker for supervisors with higher perceived status threat. Furthermore, supervisor perceived status threat moderates the relationship between GNS and innovation performance, such that this relationship becomes weaker for supervisors with higher perceived status threat. The study concludes with theoretical and practical implications, as well as future research avenues.

## Introduction

Employees differ in their level of growth need strength, which is a personality trait that refers to the needs and willingness of individuals to learn, grow, accept challenges, and achieve career development from work (Hackman and Oldham, 1976, 1980; Shalley et al., 2009). The higher the strong growth need (GNS), the more employees will pay attention to personal growth and achievement, exercise their independent thoughts, enjoy challenging work, and get more internal motivation and happiness from it (Bottger and Chew, 1986; Shalley et al., 2009; Lin et al., 2016). A secret behind a successful organization is to attract high-GNS employees and help them achieve success (Strubler and Redekop, 2010). Besides, the intensification of the global market competition and the turbulence of the organizational environment urge organizations to actively seek good development strategies. Employee innovation, under this circumstance, is the engine of organization development (Kim et al., 2013; Nieto et al., 2015). Therefore, how to improve employees’ innovation performance has become an important issue in the management field (Guo and Hu, 2022). Indeed, employees who can make intensive efforts and breakthrough attempts have good innovation performance. Obviously, employees with high GNS have the strong motivation of learning new knowledge and pursuing excellent work performance (Shalley et al., 2009) and, thus, incline to invest more energy into innovation. Thus, there should be a positive relationship between employees’ GNS and innovation performance. However, the reality is that numerous extant literature discussed the relationship between GNS and individual behaviors and attitudes (Lin et al., 2016), such as GNS and employees’ openness to experience (Graen et al., 1986), knowledge sharing behavior (Li and Ma, 2014), creativity (Shalley et al., 2009; Volmer et al., 2012), leader–member exchange (LMX) relationship quality (Phillips and Bedeian, 1994), job performance and affective commitment (Lin et al., 2016), and attitude to organizational change (Elias, 2009); little literature has verified the relationship between GNS and innovation performance. In other words, despite the natural association between GNS and innovation performance, existing studies do not explicitly validate the relationship. Hence, this paper will first discuss this research gap.

The extant literature on employee innovation mainly focuses on the internal characteristics of employees (Shalley et al., 2009), Some scholars propose that external influence of stakeholders in the organization play on employees’ innovation performance cannot be ignored either (Seibert et al., 2001; Tang and Mao, 2020). A variety of social and resource supports from stakeholders create fertile land for employees’ innovation performance. Leader, undoubtedly, is an important stakeholder. According to the leader–member dependency hypothesis, leaders need to achieve team goals through employees’ efforts, and, in turn, the feedback, guidance, and innovation resource support from leaders provide a guarantee for employees with GNS to achieve innovation performance. Consequently, there is a natural cooperative relationship between leaders and subordinates (Huang and Iun, 2006; Liu et al., 2018). We take an cooperative perspective in this study to argue that LMX has an important mediating effect on the relationship between GNS and innovation performance.

However, some contradictory phenomena cannot be explained by the leader–employee cooperation perspective. For example, a leader sometimes expresses very weak support toward subordinates with high GNS who can help him achieve team goals, and even destroy or suppress innovative behavior of subordinates with high GNS. However, the existing literature rarely discusses the phenomenon. We argue that supervisor perceived status threat can explain leaders’ “suppression” behavior possibly. As the spokesperson of the organization, a leader wants to lead team members to achieve team goals and realize personal goals. Furthermore, as rational egoists, leader’s personal goals usually are superior to team goals (Hoyos, 2013). Among the numerous personal goals of leaders, status demand is the deepest and most fundamental demand. Neuroeconomics and ecology show that people’s demand for status is overwhelming (Charness et al., 2014; Liu et al., 2015). Therefore, there is also a dynamic co-opetition relationship between leader and member from the perspective of status competition. On the one hand, leaders need to rely on the employees’ GNS to achieve team tasks (Griffin et al., 2007). On the other hand, leaders face challenges and threats brought by employees with high GNS. According to status characteristics theory (Berger et al., 1972) and social dominance theory (SDT; Sidanius et al., 2004), the leaders regard the individuals with high GNS as potential status competitors with necessary capability to obtain high status and participate in status competition. Due to the scarcity and competitiveness of organizational status resources, leaders naturally tend to protect the existing status. Leaders who perceive status threat probably make a poor response to employees’ GNS, for example, taking non-political measures to destroy the LMX relationship, and hindering the positive effect of employees’ GNS on their innovation performance. Hence, the cooperation perspective cannot effectively explain the leaders’ influence on the relationship between employees’ GNS and their innovation performance. Accordingly, this paper introduces the supervisor perceived status threat as a leadership characteristic variable to explore whether it will exert a contingency effect on the influence of employees’ GNS on LMX and innovation performance.

This study seeks to offer some contributions to the existing research literature. First, although previous studies have verified that the employees’ GNS positively affects various routine work outcomes, few studies examine the relationship between GNS and innovation performance. Our examination of whether GNS has a positive effect on innovation performance, contributes to GNS literature by providing evidence of the relationship between personality trait and innovation performance. Second, this study reveals the mediating role of LMX in the influence process of employees’ GNS on innovation performance from leader–member cooperative perspective. Our mediating approach contributes to the literature on GNS and innovation performance by revealing why and how employees’ GNS is a strong booster for their innovation performance. Third, this study provides a possible answer to an important question: under what circumstances will supervisors not promote but hinder LMX and the innovation performance of subordinates with high GNS? From the perspective of status competition, we identify supervisor perceived status threat as an important boundary condition when exploring the effects of GNS on LMX and innovation performance. Finally, the integration of leader–member cooperative perspective and the perspective of status competition in the same model is helpful to comprehensively understand the influence mechanism and boundary conditions of GNS on employees’ positive outcomes.

## Theory and Hypothesis

### Status Characteristic Theory and Social Dominance Theory

According to status characteristic theory (SCT), some dominant characteristics such as demographic variables (e.g., gender, age, seniority, race) and individual characteristics which reflect the employees’ work performance are regarded as symbols that have the potential to obtain high social hierarchy (Berger et al., 1972). SDT focuses on how culture, ideology, politics, social structure, individual psychology, and social psychology interact at different levels (Sidanius et al., 2004); the dominant high-status group suppresses the low-status group to maintain their dominance or high status (Khan et al., 2016). Grant (2013) argued that when evaluating employees’ performance, leaders should consider not only whether the employees’ behavior is needed by the organization, but also the influence of employees’ way of putting forward ideas and behavior on their status. Leaders will adopt ideas that could protect their status, identity, and honor in the organization, while ignoring or belittling suggestions that threaten their status (Hogan and Holland, 2003; Morrison and Ybarra, 2008).

On the one hand, a natural cooperative relationship exists between leaders and members (Liang et al., 2022). The subordinates with high GNS need leaders to provide resources and support for their innovative work. Leaders often rely on employees with high GNS to exert high-level innovation and initiative on work tasks to achieve team goals and performance (Volmer et al., 2012).

On the other hand, GNS as an individual characteristic variable that can improve employees’ innovation performance may be regarded by leaders as a symbol that has the potential to obtain high status and participate in the status competition (Liu et al., 2015). As a result, leaders face constant status threats and challenge from the employees with high GNS; then as conflicts and contradictions were provoked, they take defensive or non-constructive measures to resist or suppress subordinates to maintain their status (Grant et al., 2011; Chen et al., 2017; Liu et al., 2021). Therefore, supervisor perceived status threat will affect the relationship between GNS and innovation performance.

### GNS and Innovation Performance

GNS is an important variable highly related to job setting in the work characteristic model (Hackman and Oldham, 1976), reflecting the strong willingness of individuals to accept challenges, continue to learn and achieve professional development. With the widespread use of information and communication technologies (ICT) and the global popularity of COVID-19, crises and technological advances have influenced each other to bring about changes in the ways of working such as telecommuting and virtual work strategies (Abarca et al., 2020; Low et al., 2020; Garro-Abarca et al., 2021; Martínez-Navalón et al., 2021). Therefore, this also brings profound changes to the working characteristic model, such as diversity, communication, virtuality, innovation, challenge, and so on. Compared with employees with low GNS, employees with high GNS are more sensitive to new changes in work characteristics and more positively respond to them. Meanwhile, employees with high GNS take a series of proactive behaviors to seize all opportunities and even change the working environment to complete work tasks (Huselid and Day, 1991). They can be regarded as the pioneer to convey the mission of an organization, identify and solve problems. However, employees with low GNS react passively to the environment, and it is difficult for them to aware that working characteristics have changed, respond less positively, or even negatively to enriched work and challenging tasks (Lin et al., 2016). Thus, it is conjectured that employees’ GNS can positively influence their innovation performance through the following paths.

First, the generation of innovation performance encompassed various uncertainties and risks (Zhou et al., 2012). Therefore, innovation requires high concentration and initiative (Lin et al., 2016). While achieving innovation performance, employees need to have internal and continuous motivation to firmly promote themselves to face difficulties, challenges, and performance pressure. Research shows that employees with high GNS often regard complex work tasks as ideal challenges or growth opportunities, from which they can get intrinsic incentive (Hackman and Oldham, 1976) to perform more proactively in innovative work (Johnson et al., 2010). In addition, employees with high GNS generally will not passively wait for and accept everything that the environment gives. Instead, they will proactively seek and create opportunities to meet their growth needs (Shalley et al., 2009) and even modify the working environment to meet their strong demand for success (Huselid and Day, 1991). This provides motivation and opportunity basis for employees to innovate and accumulate creative output.

Second, turning creative ideas into real work results is a complex and challenging task, requiring employees to have in-depth professional knowledge and even develop and apply some new knowledge beyond their work field. Hackman and Oldham (1980) argued that employees with high GNS are more inclined to update their professional knowledge and working skills, concentrated on in-depth processing of professional knowledge, thereby further deepening their understanding of work (Wang et al., 2018). Abundant task experience and diversified knowledge improve employees’ cognitive flexibility, formulating the knowledge and ability foundation for improving innovation performance (Arias-Pére and Vélez-Jaramillo, 2022).

Third, innovation is a social activity that requires interpersonal and resource supports from the organization. Employees with high GNS actively establish relationship networks in the organization. Employees with high GNS had more knowledge-sharing behaviors on social networking sites, through which they interact with others, set up relationships and obtain social capital (Li and Ma, 2014). In addition, to promote individual growth and development, employees with high GNS build strong trust relationships with colleagues and leaders through active communication and cooperation with organization members, frequently seeking performance feedback from their supervisors. Sufficient social capital and interpersonal network form a resource base for innovation performance (Sarkawi et al., 2016). Therefore, hypothesis 1 is proposed:

*H1*: GNS is positively related to innovation performance.

### The Mediating Role of LMX

According to LMX theory, resource scarcity and subordinates’ individual differences will lead a leader to adopt different exchange strategies to establish the exchange relationship from low to high quality with member (Wilson et al., 2010). A high-quality LMX relationship is characterized by the subordinates being marked as “in-group members,” accessible to more trust, support, and preferential treatment. On the contrary, a low-quality LMX relationship equals a pure working relationship based on the power system, and subordinates, as the “out-group members” of leaders, are difficult to get extra care and rewards (Maslyn and Uhl-Bien, 2001).

As mentioned above, GNS refers to the degree to which individuals attach importance to personal growth and development opportunities at work (Oldham and hackman, 2010). Employees with high GNS focus on personal development and are willing to undertake challenging jobs (Bottger and Chew, 1986; Sarkawi et al., 2016). They are more likely to proactively seek leaders’ feedback to improve work quality continuously. During this process, employees with high GNS demonstrate the traits of a sense of responsibility, affinity, and extroversion. These characteristics incur leaders’ love, trust, and dependence, conducive to establishing a high-quality LMX relationship (Wilson et al., 2010). Moreover, employees with high GNS can provide valuable resources for leaders by imposing higher levels of innovation and initiative on tasks (Wilson et al., 2010), thus helping leaders become more effective and flexible. Previous studies have also shown that members with higher GNS are often more likely to establish a high-quality LMX relationship with their leaders (Phillips and Bedeian, 1994). Graen et al. (1986) found that the GNS of subordinates was positively correlated with the quality of LMX. Employees with high GNS have a clearer understanding of the necessity of establishing a strong network with resource controllers and better political knowledge and skills to deal with the relationship with colleagues and leaders. Therefore, it can be predicted that the higher the GNS of employees, the better they will establish high-quality exchange relationships with their leaders.

A high-quality LMX relationship provides employees with greater freedom of decision-making, broader innovation space, more innovation resource support (Zhang et al., 2012), and sufficient respect and trust (Newman et al., 2017). In addition, recognized as an “in-group member” by leaders, leaders will better understand the expectations of employees with high GNS, allocates more important organizational roles and offers more growth opportunities to them, such as more challenging work and constructive feedback and support when necessary. For subordinates, applying innovative ideas to practice encompassed a certain extent of risk and uncertainty (Yu et al., 2020). Meanwhile, subordinates will have instinctive fear and anxiety about unknown new things. However, the “in-group member” identity endowed by high-quality LMX reinforces subordinates’ sense of belonging, self-affirmation and psychological security, thus strengthening their courage to face innovation risks. They hence have more resources and motivation to carry out innovative behaviors and increase innovation performance. Therefore, the following hypothesis is proposed:

*H2*: LMX mediates the relationship between GNS and innovation performance.

### Buffering Effect of Perceived Status Threat

The status threat is defined as an individual’s perceived disrespect and denial, or an individual’ status characteristics such as official status, reputation and influence within the organization are threatened or weakened (Kramer, 1998). Due to the distinct characteristics of status resources, such as high demand, high value and strong competition (Pearce et al., 2001), position hierarchy is dynamic and unstable in specific organizational situations. Therefore, organization members always try to change or enhance their status by improving their ability, performance, and other status symbols (Berger et al., 1972).

Employees with high GNS usually focus on developing their skills and talents, possessing a strong sense of responsibility and affinity, putting forward constructive suggestions and innovative methods. Therefore, high GNS can be regarded as the potential factor and precondition for acquiring status symbols such as capability, reputation, influence, and high-performance level, making leaders often perceive status threats from subordinates (Khan et al., 2016). Furthermore, employees with high GNS are probably seen as a threat to their leaders because the employees can introduce unwelcome changes which make leaders feel embarrassed, weak and vulnerable by exposing their shortcomings and weaknesses, further doubt their incompetence (Grant and Parker, 2009). According to SDT (Sidanius et al., 2004), people with high status have a high level of social dominance. After perceiving a threat to the status in the group, leaders will amplify power and stratus differences to consolidate and maintain their own status. From the perspective of status competition, when leaders evaluate employees’ behaviors, leaders consider whether employees’ GNS is needed by the organization and whether employees’ GNS will pose a threat to his status (Ames and Flynn, 2007; Grant et al., 2011; Grant, 2013). Leaders who perceive status threats probably destroy the relationship with employees with high GNS through counterproductive political operations and adopt defensive or even exclusive communication modes toward employees, for example isolating subordinates, silent treatment, indifference, “wearing little shoes” for subordinates or deliberately concealing work information (Tang and Mao, 2020), even undertake destructive negative behaviors to deliberately provoke interpersonal conflicts in the team and viciously resist subordinates’ GNS to maintain their status. Thus LMX relationship quality is reduced. Therefore, we propose:

*H3*: Supervisor perceived status threat weakens the positive relationship between GNS and LMX.

Although the employees with high GNS can bring more innovation performance, the degree of the supervisor perceived status threat probably determines whether he is willing to provide employees with innovation support and resources. A leader with high-status threat perception will intensify his control (Liu et al., 2021) and influence to maintain his status rather than provide resources to promote employees’ innovation performance (Galinsky et al., 2010). Conversely, employees probably fail to be recognized and appreciated by the leaders because of their GNS, but they are ignored and alienated, further weakening innovation performance. Thus, we propose the following hypothesis:

*H4*: Supervisor perceived status threat weakens the positive relationship between GNS and innovation performance.

**Figure 1:**
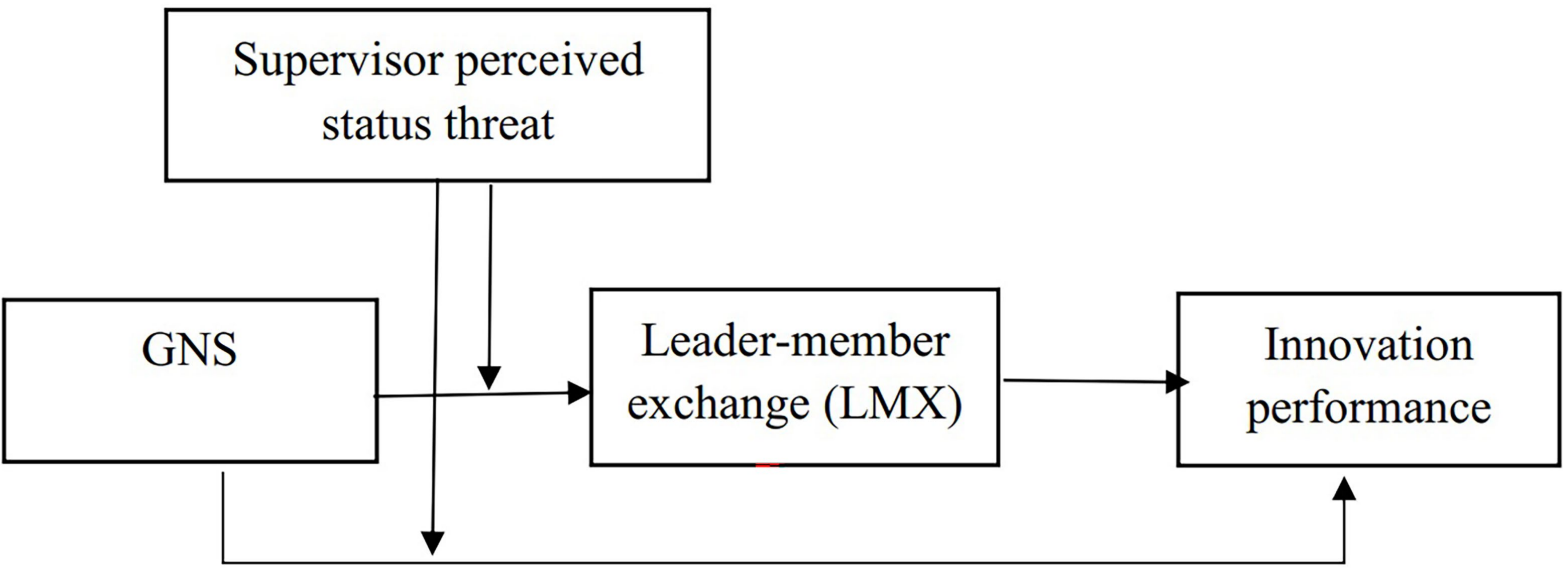
Theoretical model of the study.

The theoretical model of this study is shown in Figure 1: Theoretical model of the study.

## Materials and Methods

### Sample and Procedure

For data collection, we developed a questionnaire based on scales have been well established in relevant previous studies. High-tech enterprises have high requirements for employees’ innovation performance, and employees’ GNS can also highly explain their performance. Due to the nature of work and the short half-life of knowledge, the cooperation and competition between supervisors and subordinates are more prominent than in other industries. According to the above considerations, this study focuses on high-tech enterprises. Because of time, energy, and human resources restrictions, it was not possible to send the questionnaire to all high-tech enterprises, drawing on previous studies (e.g., Gelashvili et al., 2021), a convenience sampling approach was used and survey questionnaires were disseminated online in the Design and R&D departments of 13 high-tech enterprises from Guangzhou, Shanghai, Wuhan, and Suzhou in China. We contacted the human resources managers and arranged a formal training before their monthly meeting to briefly introduce our academic purpose and highlight the anonymity in our survey.

In order to avoid the influence of homologous bias on the research validity, the questionnaire was filled out by the supervisor and the subordinates in pairs. The employee’s direct supervisor evaluated the employee’s innovation performance and GNS, and self-reported perceived status threat and GNS. The subordinate self-reported GNS, LMX. Employees’ GNS was reported by supervisors and subordinates at the same time, the samples with great variation in the results reported by both parties were eliminated [the difference (absolute value) of the results was greater than or equal to 3] to minimize the possible error caused by social approval, subordinates’ self-evaluation of GNS data was retained. The supervisor and subordinate questionnaire adopt a ratio of 1:3, meaning that one supervisor only randomly evaluates three subordinates in the team. The data were collected in two stages with the consideration of the lag of employees’ innovation performance. In the first stage (Time 1), GNS, LMX, and perceived status threats were collected. The second stage (Time 2) survey was conducted to assess employees’ innovation performance 2 months later. This study carried out anonymous processing to protect the privacy of participants. Each participant was given a serial number, through which the data collected in the two stages were combined into complete data. A total of 239 sets of questionnaires were distributed to nearly 90 different teams in 13 companies. In the end, we received 210 sets of valid questionnaire, for an effective rate of 87.9%.

Among 210 supervisor questionnaires, 49.05% were female. The average age was 38.8 years old and mainly distributed between 28 and 45 years old. Most had advanced degree: 56.12% had a bachelor’s degree and 22.31% had a master’s degree or above. The average organizational tenure was 8.18 years, and the average work time with subordinate was 4.24 years. Among 630 subordinate questionnaires, 46.51% were females. The average age was 25.16 years old and mainly distributed between 22 and 35 years old. 64.11% had a bachelor’s degree and 26.62% had a master’s degree or above. The average organizational tenure was 4.17 years, and the work time with the supervisor was 3.1 years.

### Measures

All scales used in this research have been well established in the literature to ensure rigor and credibility and have been revised according to the actual situation in China. Back translation was performed to avoid semantic confusion affecting the quality of the questionnaire (Brislin, 1970). First, two doctoral students who majored in human resource management translated the English version of the survey into Chinese. Second, the two students exchanged the Chinese version and translated it back into English. Third, they discussed and modified the Chinese version according to the back translation. Finally, two professors verified the surveys using their professional experience to ensure that the final Chinese version was clear to understand. All scales in this study were measured on a five-point Likert scale from 1 (strongly disagree) to 5 (strongly agree).

*GNS(Time 1)* GNS was measured using seven-item scale developed by Hackman and Oldham (1980). By referring to the practice of Heckler (1996), finally, 5 items were retained after eliminating 2 items with a factor load less than 0.5. Some of the items used were, “I will exert my imagination and creativity in my work,” “I will look for opportunities for personal growth and development.” The Cronbach’s alpha of the GNS scale was 0.838.

*Innovation performance(Time 2)* The scale of innovation performance was adapted from a scale with 5 items developed by Zhou and George (2001). The employee’s innovation performance was measured by the direct supervisor because previous study showed that supervisor evaluation was more reliable than the subordinate evaluation (George and Zhou, 2001). Some of the items used were, “I often put forward some new methods and suggestions to improve the work results or product quality,” “I often adopt new methods to solve problems in work.” The Cronbach’s alpha for the innovation performance scale was 0.901.

*LMX(Time 1)* LMX was measured using the one-dimensional scale proposed by Graen and Uhl-Bien (1995), including 7 items, Some of the items used were, “I think the relationship between me and my supervisors is harmonious,” “When I encounter difficulties in work, I believe my supervisors can help me solve the problem together.” The Cronbach’s alpha for the LMX scale was 0.891.

*Supervisor perceived Status Threat (Time 1)* The supervisor perceived status threat scale was adapted from the scale used to measure the perceived status threat of team members from Okimoto and Wenzel (2011). There are 3 items in total. “Some of the subordinate’s work practices weaken your status in the organization,” “Some of the subordinate’s work practices make you feel disrespected in the organization,” and “Some of the subordinate’s work practices make you feel you are being questioned in the organization.” The Cronbach’s alpha for this scale was 0.837.

*Control Variable(Time 1)* We controlled the four demographic variables of age, education level, organizational tenure and working years with supervisor. Because employees’ age and education level were closely related to employees’ GNS and innovation performance (Hackman and Oldham, 1980; Lin et al., 2016), the organizational tenure and working years with supervisors would affect the LMX (Zhang et al., 2012), further affecting employees’ GNS and innovation performance.

### Analytical Strategy

We first examine the distinctiveness of the research variables, and we conducted a confirmatory factor analysis (CFA) using Amos 23.0 to compare the fit of our hypothesized four-factor model to the fit of alternative models.

Moreover, as we proposed direct effect (i.e., Hypothesis 1), indirect effect (i.e., Hypothesis 2) and moderating effect (i.e., Hypothesis 3 and Hypothesis 4), we employed the hierarchical regressions to examine the proposed direct effect, indirect effect and interactive effects. Specially, we required the following conditions for mediation: (a) the independent variable must be related to the mediator; (b) the mediator must be related to the dependent variable; and (c) the independent variable must have no effect on the dependent variable when the mediator is held constant (full mediation), or the effect should become significantly smaller (partial mediation) (Kenny et al., 1998). To further assess the mediating hypothesis, we assessed the indirect effects with the bootstrapping technique using SPSS 24.0.

Besides, we followed Aiken et al. (1991) recommendation for plotting the interactions.

## Results

### Confirmatory Factor Analysis

CFA was used to test the discriminant validity of the four key variables: GNS, LMX, supervisor perceived status threat, and innovation performance. All variables were analyzed directly in the items (Netemeyer et al., 1990). Against the baseline model of four factors, five alternative models were examined. Table 1 presents the results of CFA. The proposed fit indices of four-factor model (*χ*^2^ = 526.38, df = 203, NFI = 0.93, TLI = 0.91, CFI = 0.92, GFI = 0.94, RMSEA = 0.06) is significantly better than the three-factor model, two-factor model, and one-factor model, The results indicate that the four-factor model was better than any of the alternatives, indicating good discriminant validity between each variable.

**Table 1:** Results of confirmatory factor analysis (CFA).

Models	Factor structures	*χ* ^2^	*χ*^2^/df	NFI	TLI	GFI	CFI	RMSEA
Four-factor	GNS; LMX; innovation performance; perceived status threat	526.38	203	0.93	0.91	0.94	0.92	0.06
Three-factor 01	GNS and LMX combined	965.17	206	0.83	0.79	0.68	0.78	0.14
Three-factor 02	LMX and innovation performance combined	1134.67	206	0.68	0.70	0.62	0.76	0.16
Two-factor 01	GNS and LMX combined; innovation performance and perceived status threat combined	1300.55	208	0.65	0.67	0.59	0.69	0.15
Two-factor 02	GNS and innovation performance combined; LMX and perceived status threat combined	1247.65	208	0.68	0.72	0.68	0.72	0.15
One-factor	All factors combined into one factor	1701.29	209	0.53	0.5	0.57	0.60	0.19

**n* = 210; TLI is the Tucker–Lewis index; GFI is the goodness-of-fit index; CFI is the comparative fit index; NFI is thenormed fit index; and RMSEA is the root mean square error of approximation*.

### Descriptive Analysis

Table 2 presents the descriptive statistics and zero-order correlations of the variables and includes GNS, LMX, supervisor perceived status threat, and innovation performance. As expected, GNS are significantly positively correlated with innovation performance (*r* = 0.45, *p <* 0.01), LMX (*r* = 0.24, *p <* 0.01), and supervisor perceived status threat (*r* = 0.19, *p <* 0.01). LMX are significantly positively correlated with innovation performance (*r* = 0.34, *p <* 0.01). The correlation coefficients confirm our hypotheses. Additionally, education level is positively related to GNS (*r* = 0.20, *p <* 0.01), and innovation performance (*r* = 0.09, *p <* 0.05), is negatively related to LMX (*r* = −0.09, *p <* 0.05). Organizational tenure is positively related to innovation performance (*r* = 0.02, *p <* 0.05). Years of working with supervisors was negatively related to GNS (*r* = −0.08, *p <* 0.05) and was positively related to innovation performance (*r* = 0.02, *p <* 0.05).

**Table 2:** Descriptive statistics and zero-order correlations.

Variable	*M*	*SD*	1	2	3	4	5	6	7
1. Age	29	5.51							
2. Education	2.78	0.79	−0.01						
3. Organizational tenure	5.91	0.94	0.05^**^	−0.01^*^					
4. Years of working with supervisors	2.66	1.24	0.09	0.03	0.06^*^				
5. GNS	3.62	1.04	0.04	0.20^**^	−0.03	−0.08^*^			
6. LMX	3.39	0.94	0.04	−0.09^*^	−0.05	0.07	0.24^**^		
7. Innovation performance	3.58	1.04	0.08	0.09^*^	0.02^*^	0.02^*^	0.45^**^	0.34^**^	
8. Perceived status threat	2.81	1.06	−0.03	0.06	−0.03	0.02	0.19^**^	−0.19^**^	−0.27^**^

*n = 210. LMX, Leader–Member Exchange; GNS, Growth Need Strength*.

* *p < 0.05 and*

** *p < 0.01*.

### Hypotheses Testing

The hypotheses were tested using Mplus 7.0. The coefficient results are shown in Table 3.

**Table 3:** Results of the hypothesis test.

	LMX			Innovation performance		
	M1	M2	M3	M4	M5	M6
Age	−0.06	−0.05	0.05	0.08	0.07	0.07
Education	0.07	0.09	0.09^*^	0.10^*^	0.08^*^	0.02^*^
Organizational tenure	0.05	0.20	0.05	−0.06	−0.03	0.07
Years of working with supervisors	0.09	0.09^*^	0.02^*^	0.07	0.07	0.05
GNS	0.39^***^	0.29^***^	0.44^***^		0.36^***^	0.40^***^
LMX				0.48^***^	0.33^***^	
Perceived status threat		−0.22^***^				−0.22^**^
GNS*Perceived status threat		−0.34^***^				−0.25^**^
*R* ^2^	0.29	0.41	0.23	0.32	0.33	0.31
*R*^2^ Change	0.24	0.12	0.18	0.27	0.10	0.08
*F*	44.15^***^	21.56^***^	39.15^***^	40.60^***^	49.12^***^	16.68^***^

*n = 210. LMX*, *Leader–Member Exchange; GNS, Growth Need Strength*.

* *p < 0.05,*

** *p < 0.01, and*

*** *p < 0.001*.

First, the direct effect of employees’ GNS on innovation performance was tested. The analysis results show that GNS has a significant positive effect on innovation performance (M3, *β* = 0.44, *p* < 0.001), and Hypothesis 1 is supported.

Second, the mediating effect of LMX was tested. According to the regression results of M1 and M4, GNS has a significant positive effect on LMX (M1, *β* = 0.39, *p* < 0.001), and LMX has a positive effect on innovation performance (M4, *β* = 0.48, *p* < 0.001). The GNS and LMX are entered into the regression model, the LMX positively correlates with innovation performance (M5, *β* = 0.33, *p* < 0.001), but the positive effect of GNS on innovation performance is reduced (M5, *β* = 0.36, *p* < 0.001). The results show that the LMX plays a partial mediating role between GNS and innovation performance (Kenny et al., 1998), and Hypothesis 2 is supported. Hypothesis 2 was also tested using bootstrap resampling (5,000 times), which allows us to see the algebraic sign, the magnitude and the significance of the hypotheses put forward (Martínez-Navalón et al., 2021). If the confidence intervals of the results exclude 0, the mediation effect is supported (Preacher et al., 2010). The result shows that the mediation effect is 0.246 with a 95% confidence interval of [LLCT = 0.1713, ULCI = 0.2845], not including 0. Therefore, GNS had a positive effect on innovation performance indirectly through LMX. Besides, after controlling the mediating variable LMX, the independent variable GNS has a significant effect on the dependent variable innovation performance with a 95% confidence interval of [LLCT = 0.4137, ULCI = 0.5864]. Therefore, LMX plays a partial mediating role between employees’ GNS on their innovation performance, supporting Hypothesis 2.

**Figure 2:**
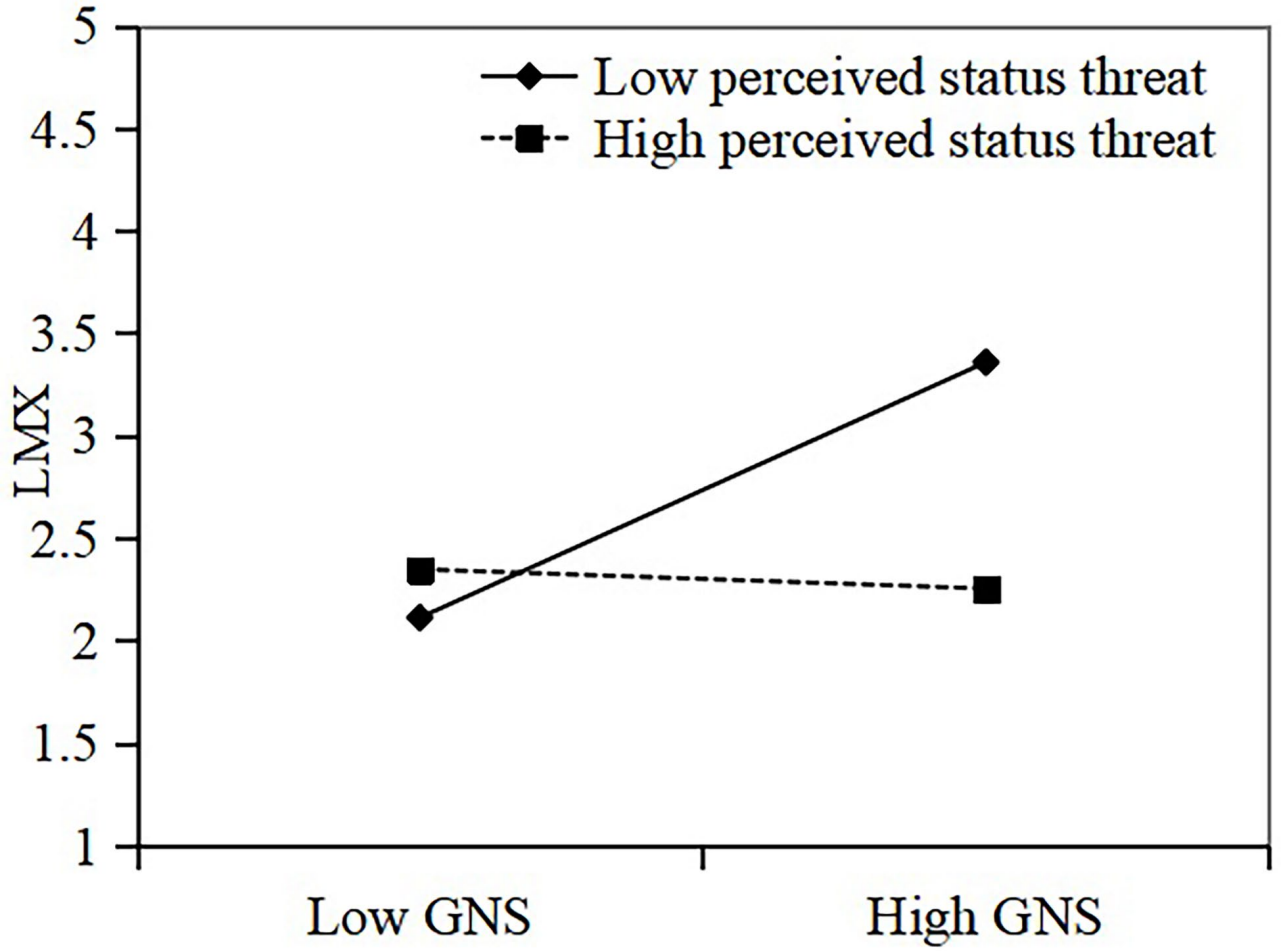
Interaction impacts of GNS and perceived status threat on LMX.

**Figure 3:**
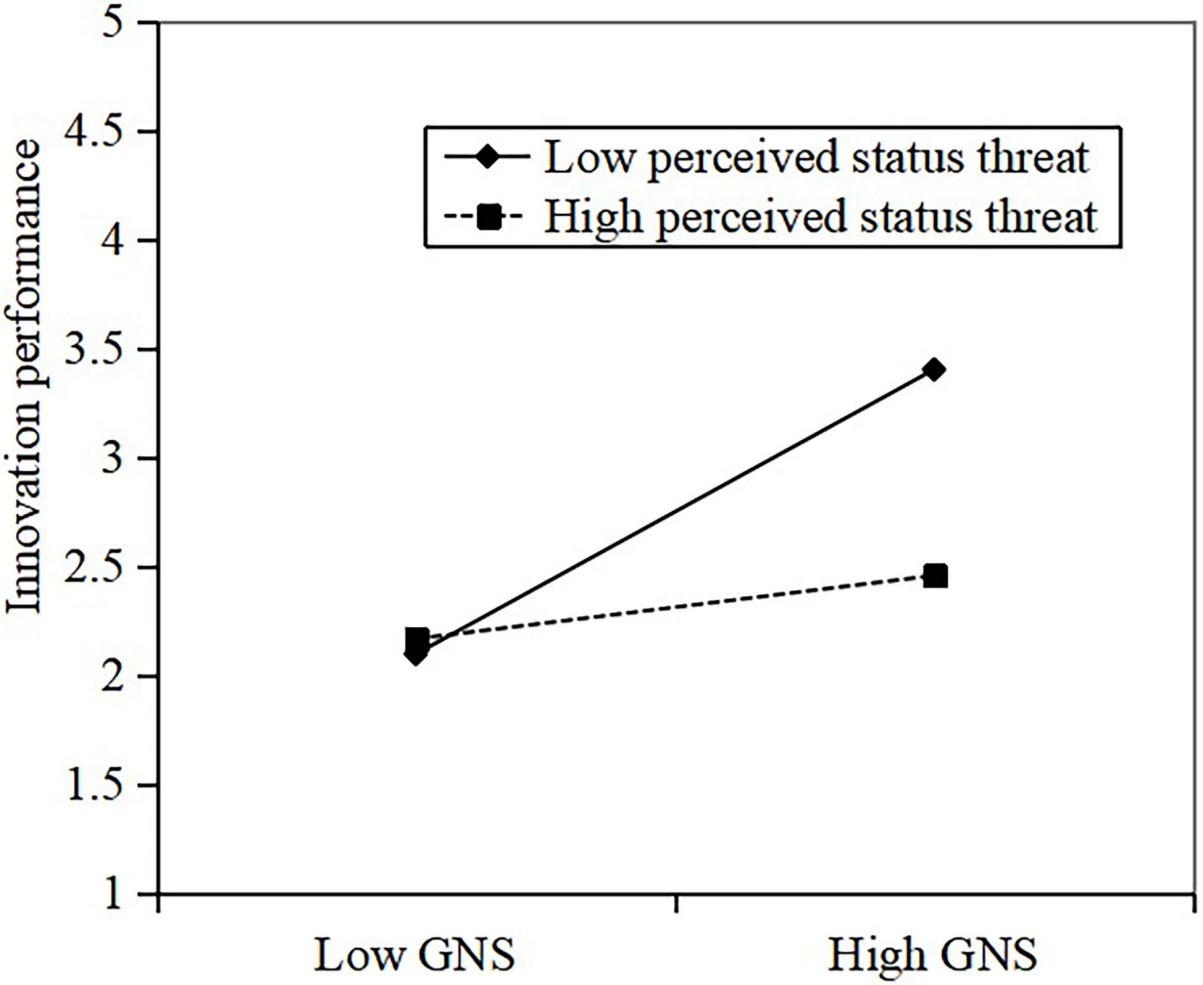
Interaction impacts of GNS and perceived status threat on innovation performance.

Finally, hierarchical regression analyses to test our hypotheses regarding the moderating effect of supervisor perceived status threat on the relationship between GNS and LMX and the relationship between GNS and innovation performance. To minimize any potential problems with multicollinearity, we centered the predictor variables before calculating the interaction terms (Aiken et al., 1991). As shown in Table 3, the interaction between GNS and supervisor perceived status threat on LMX is significant (M2, *β* = −0.34, *p* < 0.001), indicating that supervisor perceived status threat has a significant moderating effect on the relationship between GNS and LMX. Hypothesis H3 is supported. Besides, a simple slope test suggests that the relationship between GNS and LMX is significantly positive when the supervisor perceived status threat is low (see Figure 2). When the supervisor perceived status threat is high, the relationship between GNS and LMX is weak. As shown in Table 3, the interaction between GNS and supervisor perceived status threat on innovation performance was significant (M6, *β* = −0.25, *p* < 0.01), indicating that supervisor perceived status threat had a negative moderating effect on the relationship between GNS and innovation performance. Thus, Hypothesis 4 was supported. The interaction effect of GNS and perceived status threat on innovation performance is shown in Figure 3. Compared with low supervisor perceived status threat, the positive relationship between NGS and innovation performance decreases when the supervisor perceived status threat is high.

## Discussion

This study constructed a new theoretical model from the perspective of status competition to explore the significant positive effect of employees’ GNS on innovation performance *via* LMX, and the boundary conditions of supervisor perceived status threat. This study found that employees’ GNS significantly affects their innovation performance. This result coincides with other previous findings that confirm that GNS can lead to positive working outcomes such as knowledge sharing behavior (Li and Ma, 2014), job performance and organizational affective commitment (Lin et al., 2016), and organizational identification (Wang and Yang, 2015), which has increased our knowledge about the outcomes of GNS by explicitly validate the positive relationship between GNS and innovation performance.

Furthermore, the results confirmed that LMX plays a mediating role between GNS and innovation performance, that is, employees’ GNS affects their innovation performance by influencing LMX. This is consistent with leader–member dependency hypothesis and leader–member cooperative perspective (Huang and Iun, 2006; Liu et al., 2018; Li and Huang, 2021). Although research has explored the mediating role of personal emotions and attitudes in the relationship between GNS and employee outcomes, little research has specifically examined how GNS affects innovation performance from the leader–member cooperative perspective. For example, Lin et al. (2016) showed hope mediates the effect of growth need strength on job performance and affective commitment based on hope theory. Li and Huang (2021) stated implicitly that LMX may play a mediating role in predicting the relationship between personality trait and innovation performance. Thus, consistent with past research, we have found that GNS causes reciprocity and cooperation between supervisors and subordinates, leading to high-quality LMX and resulting in innovation performance. We thus contribute to GNS literature by providing evidence that facilitates the understanding of the relationship between GNS and positive employee outcomes through LMX.

Different from previous studies, our research has proposed and found that supervisor perceived status threat played a consistent negative moderating role on the relationship between employees’ GNS and innovation performance and between employees’ GNS and LMX. From the perspective of status competition, this paper explained how leaders’ psychological state and behavior choice affect the relationship between GNS and its positive results. This contributes to GNS literature by enhancing our understanding of the boundary conditions of GNS on employees positive outcomes. Existing researches have mainly focused on supportive or cooperative perspective to emphasize that the leadership style (Gu et al., 2015), LMX (Pan et al., 2012) promoted employees’ innovative behavior or innovation performance. There is no denying that these studies do make a significant contribution in exploring how leadership characteristics or behavior affect innovation performance. However, the existing studies cannot explain some special phenomena in reality, for example, why do the supervisors weakly support their subordinates with high GNS who can obviously help them achieve goals? What is the deep mechanism of action? In the present study, we argued that status competition between supervisors and subordinates can answer these questions, our research has proposed and found that that supervisor perceived status threat buffers the positive effect of GNS on LMX and innovation performance. On the one hand, according to status characteristics theory and SDT, supervisors who perceived status threat may destroy the LMX relationship with employees with high GNS through counterproductive political operations. On the other hand, in order to maintain the existing status or to reduce the status threat, those supervisors who perceived status threat from the employees with high GNS will take undermining behavior such as hiding the information that needed by employees with high GNS or reduce the support or help to them, finally reduce their innovation performance (Duffy et al., 2002; Chen et al., 2017; Liu et al., 2021). Thus, our research enhances the current knowledge about how leaders’ psychological state and behavior responses affect the relationship between GNS and innovation performance.

## Conclusion

The findings of our study reveal that employees’ GNS is positively related to their innovation performance. In addition, LMX plays a significant mediation role in transmitting the effect of GNS to innovation performance. Finally, we found that supervisor perceived status threat moderated the relationship between GNS and LMX, such that this relationship got weaker for supervisors with higher perceived status threat. Furthermore, supervisor perceived status threat moderated the relationship between GNS and innovation performance, as such, it became weaker for supervisors with higher perceived status threat. By examining the joint effect of GNS and supervisor perceived status threat on GNS and innovation performance, we have enhanced the understanding of how leaders’ psychological state and behavior choice affect the relationship between GNS and its positive results. Therefore, we recommend that organizations and supervisors identify employees’ growth need strength and help them develop LMX by adopting appropriate leadership styles and reducing supervisor perceived status threat. As a result, employees will generate more innovation performance to the organization and become better performers.

### Theoretical Contributions

First, our study explored the significant positive effect of employees’ GNS on their innovation performance from the perspective of employees’ needs level and expanded the research on the antecedent variables of innovation performance.

Second, our paper identified LMX as an interpersonal relationship mechanism effectively mediating the relationship between GNS and innovation performance. The employees’ GNS can significantly promote their exchange relationship with leaders, while LMX promotes employees’ innovation performance. Although scholar stated that LMX may play a mediating role in the relationship between personality trait and innovation performance from leader–member cooperative perspective (e.g., Li and Huang, 2021), there is still a lack of relevant empirical support for this view. Our study provides early empirical evidence to echo calls for examining the mediating role of LMX. Thus, our study clarifies the influence mechanism of GNS on innovation performance.

Finally, we incorporate SCT and SDT, into organizational management research and propose supervisor perceived status threat play a consistent negative moderating role on the relationship between employees’ GNS and innovation performance and between employees’ GNS and LMX. Our study, from the perspective of status competition, clarifies that supervisor perceived status threat was an important boundary condition between GNS and innovation performance, answers the question that why supervisors sometimes expresses very weak support toward subordinates with high GNS. Our study goes one step further and takes a new theoretical research perspective of the roles of growth need strength in employees’ positive outcomes.

### Managerial Implications

This study helps to understand GNS from the perspective of status competition deeply and puts forward a new perspective on how management practices can improve employees’ innovation performance.

First, our study emphasizes that employees with high initiative (e.g., growth need strength) are the key resources for personal innovation and organizational success. Therefore, the cornerstones of human resource activities are to getting, keeping, and growing such employees for organizations. Further, we suggest that human resource management practices should prioritize finding each employee’s GNS and focus on enhancing and developing employees’ capabilities, wellness, and prosperity. To assess an employee’s level of growth need strength, organizations could use a survey questionnaire developed by organizational studies (e.g., Hackman and Oldham, 1980) or a clinical classification developed by positive psychology research. More importantly, we suggest that human resource managers and supervisors should communicate with individual employees and observe their behavior and attitude to identify the employees with high internal expectations and desires for accomplishment, learning, and personal development within their jobs, and then, to provide them with support and resources to improve their innovation performance.

Second, our finding suggests that employees fulfill their growth need and achieve innovation performance *via* LMX. Organizations should help employees develop high-LMX by adopting appropriate leadership styles and considering each employee’s personality characteristics. On the one hand, we suggest employees should actively seek feedback and guidance from leaders. When the organization encounters difficulties or challenges, employees should show initiative in solving problems independently and unconventionally, or communicate with the leader and make suggestions when necessary. On the other hand, supervisor should pay more attention to the requirements and expectations of their employees, and also be sensitive to employees’ emotional states and innovative needs, if they find that an employee with high GNS is suffering from innovation risk and uncertainty, an additional management action, such as timely communication and work lightening, may help to reinforce subordinates’ sense of belonging, self-affirmation and psychological security, thus strengthening their courage to face innovation risks. Thus, a LMX with high-quality will be establish to improve employees’ innovation performance.

Third, our finding regarding a negative moderating effect of supervisor perceived status threat shows that the status competition have significant influences on the impact of GNS. The findings of this study sounded an alarm for managers. Although employees’ GNS can bring high innovation performance, those employees who have high GNS but lack accurate judgment over the supervisor perceived status threat will not be loved and supported by their leaders. Therefore, the organization should design a scientific and reasonable incentive mechanism to reduce the psychological defense and negative behavior of leaders. Besides, the organization should attach importance to the selection and training of grassroots leaders and implement the recruitment standards of “integrity and ability” for leading cadres.

### Limitations and Future Research Directions

Despite its contributions, this study does have its share of limitations. First, the supervisor completes the evaluation of employees’ innovation performance with some intentional or unintentional subjective deviations. In the future, objective indicators such as the number of patents, innovation awards, and the number of innovative proposals adopted can be considered for measurement. Second, from the perspective of status competition, this study verifies that the supervisor perceived status threat is an important boundary condition for the effect of GNS on innovation performance. The uncertainty of leadership power and status is probably another important boundary condition. When leaders have high “reference power” and high achievement or status, no matter how much status threat employee with high GNS brings to them, leaders will be open to employees with high GNS because leaders feel that their strong status and authority will not be threatened. Third, although our research examined the relationship between GNS, LMX, supervisor perceived status threat, and innovation performance in a non-Western culture (i.e., China), we did not provide much information about whether this relationship would be different across varying cultures. For example, since employees with high power distance obey supervisors’ expectation unconditionally, it may be possible that this relationship will be weaker in low power distance culture. Thus, it is worthwhile for future researchers to conduct cross-culture comparison study to examine whether there is a culture difference.

## Data Availability Statement

The data analyzed in this study is subject to the following licenses/restrictions: The dataset involves personal privacy. Requests to access these datasets should be directed to 25690401@qq.com.

## Author Contributions

YT and ZD designed and adopted the study, and wrote the paper. XH and RT wrote the paper. All authors contributed to the article and approved the submitted version.

## Funding

This research was funded by National Natural Science Foundation of China (no. 72062006); National Natural Science Foundation Youth Project of Guangxi Zhuang Autonomous region (no. 2019JJB180009); Philosophy and social science program of Guangxi Zhuang Autonomous region (no. 20FGL013); and Scientific Research Fund of Pearl River-Xijiang Economic Belt Development Research Institute (no. ZX2020006).

## Conflict of Interest

The authors declare that the research was conducted in the absence of any commercial or financial relationships that could be construed as a potential conflict of interest.

## Publisher’s Note

All claims expressed in this article are solely those of the authors and do not necessarily represent those of their affiliated organizations, or those of the publisher, the editors and the reviewers. Any product that may be evaluated in this article, or claim that may be made by its manufacturer, is not guaranteed or endorsed by the publisher.

## References

[ref1] Abarca V. Palos-Sanchez P. R. Rus-Arias E. (2020). Working in virtual teams: a systematic literature review and a bibliometric analysis. IEEE Access 8, 168923–168940. doi: 10.1109/ACCESS.2020.3023546

[ref2] Aiken L. S. West S. G. Reno R. R. (1991). Multiple Regression: Testing and Interpreting Interactions. London: Sage

[ref3] Ames D. R. Flynn F. J. (2007). What breaks a leader: The curvilinear association between assertiveness and leadership. J. Pers. Soc. Psychol. 92, 307–324. doi: 10.1037/0022-3514.92.2.307, PMID: 17279851

[ref4] Arias-Pére J. Vélez-Jaramillo J. (2022). Ignoring the three-way interaction of digital orientation, not-invented-here syndrome and employee’s artificial intelligence awareness in digital innovation performance: a recipe for failure. Tech. Forecas. Soc. Change 174:121305. doi: 10.1016/J.TECHFORE.2021.121305

[ref5] Berger J. Cohen B. P. Zelditch M. (1972). Status characteristics and social interaction. Am Sociol Rev 37, 241–255. doi: 10.2307/2093465

[ref6] Bottger P. C. Chew I. K. (1986). The job characteristics model and growth satisfaction: main effects of assimilation of work experience and context satisfaction. J. Human Relat. 39, 575–594. doi: 10.1177/001872678603900606

[ref7] Brislin R. W. (1970). Back-translation for cross-cultural research. J. Cross-Cultural Psychol. 1, 185–216. doi: 10.1177/135910457000100301

[ref8] Charness G. Masclet D. Villeval M. C. (2014). The dark side of competition for status. J. Manage. Sci. 60, 38–55. doi: 10.1287/mnsc.2013.1747

[ref9] Chen W. Y. Ye M. L. Chen Y. S. Peng J. (2017). Does subordinate creative deviance evoke supervisor undermining? The roles of perceived threat to hierarchy and authoritarianism. J. Psychol. Sci. 40, 670–677. doi: 10.16719/j.cnki.1671-6981.20170325

[ref10] Duffy M. K. Ganster D. C. Pagon M. (2002). Social undermining in the workplace. J. Acad. Manage. 45, 331–351. doi: 10.2307/3069350

[ref500] Elias S. M. (2009). Employee commitment in times of change: assessing the importance of attitudes toward organizational change. J. Manage. 35, 37–55. doi: 10.1177/0149206307308910

[ref11] Galinsky A. D. Magee J. C. Ena L. M. Gruenfeld D. H. (2010). Power and perspectives not taken. J. Psychol. Sci. 17, 1068–1074. doi: 10.1111/j.1467-9280.2006.01824.x 17201789

[ref12] Garro-Abarca V. Palos-Sanchez P. Aguayo-Camacho M. (2021). Virtual teams in times of pandemic: factors That influence performance. Front. Psychol. 12:624637. doi: 10.3389/fpsyg.2021.624637, PMID: 33679543 PMC7925899

[ref13] Gelashvili V. Martínez-Navalón J. G. Saura J. R. (2021). Using partial least squares structural equation modeling to measure the moderating effect of gender: An empirical study. J. Undergrad. Math. 9:3150. doi: 10.3390/MATH9243150

[ref14] George J. M. Zhou J. (2001). When openness to experience and conscientiousness are related to creative behavior: an interactional approach. J. Appl. Psychol. 86, 513–524. doi: 10.1037/0021-9010.86.3.513, PMID: 11419810

[ref15] Graen G. B. Scandura T. A. Graen M. R. (1986). A field experimental test of the moderating effects of growth need strength on productivity. J. Appl. Psychol. 71, 484–491. doi: 10.1037/0021-9010.71.3.484

[ref16] Graen G. B. Uhl-Bien M. (1995). Relationship-based approach to leadership: development of leader-member exchange (LMX) theory of leadership over 25 years: applying a multi-level multi-domain perspective. J. Lead. G. 6, 219–247. doi: 10.1016/1048-9843(95)90036-5

[ref17] Grant A. M. (2013). Rocking the boat but keeping it steady: the role of emotion regulation in employee voice. J. Acad. Manage. 56, 1703–1723. doi: 10.5465/amj.2011.0035

[ref18] Grant A. M. Gino F. Hofmann D. A. (2011). Reversing the extraverted leadership advantage: the role of employee proactivity. J. Acad. Manage. 54, 528–550. doi: 10.5465/AMJ.2011.61968043

[ref19] Grant A. M. Parker S. K. (2009). Redesigning work design theories: the rise of relational and proactive perspectives. J. Acad. Manage. 3, 317–375. doi: 10.1080/19416520903047327

[ref20] Griffin M. A. Neal A. Parker S. K. (2007). A new model of work role performance: positive behavior in uncertain and interdependent contexts. J. Acad. Manage. 50, 327–347. doi: 10.2307/20159857

[ref21] Gu Q. Tang T. Wan J. (2015). Does moral leadership enhance employee creativity? Employee identification with leader and leader–member exchange (lmx) in the chinese context. J. Bus. Ethics 126, 513–529. doi: 10.1007/s10551-013-1967-9

[ref22] Guo S. H. Hu Q. Q. (2022). Energetic learning: the effect of organizational identification and thriving at work on innovation performance. J. Manage Rev. 34, 205–217. doi: 10.14120/j.cnki.cn11-5057/f.2022.01.011

[ref23] Hackman J. R. Oldham G. R. (1976). Motivation through the design of work: test of a theory. J. Organ. Beha. Human Perform. 16, 250–279. doi: 10.1016/0030-5073(76)90016-7

[ref24] Hackman J. R. Oldham G. R. (1980). Work Redesign. Addison-Wesley; Boston.

[ref25] Heckler C. E. (1996). A step-by-step approach to using the SASTM system for factor analysis and structural equation modeling. J. Tech. 38, 296–297. doi: 10.1080/00401706.1996.10484524

[ref26] Hogan J. Holland B. (2003). Using theory to evaluate personality and job performance relations: a socioanalytic perspective. J. Appl. Psychol. 88, 100–112. doi: 10.1037/0021-9010.88.1.100, PMID: 12675398

[ref27] Hoyos L. (2013). Cooperation, solidarity and rational egoism. Regarding the relationship between morality and rationality. J. Revista De Estudios Sociales 57, 24–30. doi: 10.7440/res46.2013.03

[ref600] Huang X. Iun J. (2006). The impact of subordinates-supervisor similarity in growth need strength on work outcomes: the mediating role of perceived similarity. J. Organ. Behav. 8, 1121–1148. doi: 10.1002/job.415, PMID: 24117806

[ref28] Huselid M. A. Day N. E. (1991). Organizational commitment, job involvement, and turnover: a substantive and methodological analysis. J. Appl. Psychol. 76, 380–391. doi: 10.1037/0021-9010.76.3.380

[ref29] Johnson R. E. Chang C. H. Yang L. Q. (2010). Commitment and motivation at work: the relevance of employee identity and regulatory focus. J. Acad. Manage. Rev. 35, 226–245. doi: 10.5465/AMR.2010.48463332

[ref30] Kenny D. A. Kashy D. A. Bolger N. (1998). “Data analysis in social psychology,” in Handbook of Social Psychology. eds. Gilbert D. T. Fiske S. T. Lindzey G.. 4th Edn. (New York, NY: McGraw-Hill), 233–265.

[ref31] Khan A. K. Moss S. Quratulain S. Hameed I. (2016). When and how subordinate performance leads to abusive supervision: a social dominance perspective. J. Manage. 44, 2801–2826. doi: 10.1177/0149206316653930

[ref32] Kim S. K. Arthurs J. D. Sahaym A. Cullen J. B. (2013). Search behavior of the diversified firm: the impact of fit on innovation. J. Strat. Manage. 34, 999–1009. doi: 10.1002/smj.2038

[ref33] Kramer R. M. (1998). Paranoid cognition in social systems: thinking and acting in the shadow of a doubt. J. Person. Soc. Psychol. Rev. 2, 251–275. doi: 10.1207/s15327957pspr0204_3, PMID: 15647133

[ref34] Li L. L. Huang G. (2021). “Advantages and disadvantages” of individual proactive behavior in organizations. J. Adv. Psychol. Sci. 29, 1484–1496. doi: 10.3724/SP.J.1042.2021.01484

[ref35] Li S. M. Ma W. W. K. (2014). Exploring interpersonal relationship and growth need strength on knowledge sharing in social media. Lect. Notes Comput. Sci 8595, 288–299. doi: 10.1007/978-3-319-08961-4_27

[ref36] Liang Y. Liu Y. Park Y. Wang L. (2022). Treat me better, but is it really better? Applying a resource perspective to understanding leader-member exchange (LMX), LMX differentiation, and work stress. J. Occup. Health Psychol. 27, 223–239. doi: 10.1037/ocp0000303, PMID: 34807679

[ref38] Lin X. S. Qian J. Li M. Chen X. C. (2016). How does growth need strength influence employee outcomes? The roles of hope, leadership, and cultural value. Int. J. Human Res. Manage. 29, 2524–2551. doi: 10.1080/09585192.2016.1255901

[ref39] Liu Z. Q. Li C. Liao J. Q. Long L. R. (2015). The individual status in organizations, the status-conferral ways and employee’s creative outcomes: a case study made from state-owned enterprises in China. J. Management World 3, 86–101–187–188. doi: 10.19744/j.cnki.11-1235/f.2015.03.009

[ref40] Liu Z. Q. Wei L. H. Wang F. J. Tang S. S. (2018). Growth need strength of supervisors & subordinates, incentive structure and employee creative outcomes. J. Manage. World 9, 95–108. doi: 10.19744/j.cnki.11-1235/f.2018.09.009

[ref41] Liu W. X. Zhu Y. H. Wang H. J. Liu W. D. (2021). Research on the impact of followers’s upward dissent and supervisor underming from the hierarchical threat perspective. Chinese J. Manage. Manage. 19, 197–204. doi: 10.3969/j.issn.1672-884x.2022.02.005

[ref42] Low D. M. Rumker L. Talkar T. Torous J. Ghosh S. S. (2020). Natural language processing reveals vulnerable mental health support groups and heightened health anxiety on Reddit during COVID-19.: observational study. J. Med. Int. Res. 22:e22635. doi: 10.31234/osf.io/xvwcy, PMID: 32936777 PMC7575341

[ref43] Martínez-Navalón J. G. Gelashvili V. Gómez-Ortega A. (2021). Evaluation of user satisfaction and trust of review platforms: analysis of the impact of privacy and E-WOM in the case of TripAdvisor. Front. Psychol. 12:750527. doi: 10.3389/fpsyg.2021.750527 34603166 PMC8483177

[ref44] Maslyn J. Uhl-Bien M. (2001). Leader-member exchange and its dimensions: effects of self-effort and other’s effort on relationship quality. J. Appl. Psychol. 86, 697–708. doi: 10.1037/0021-9010.86.4.697, PMID: 11519653

[ref45] Morrison K. R. Ybarra O. (2008). The effects of realistic threat and group identification on social dominance orientation. J. Expe. Soc. Psychol. 44, 156–163. doi: 10.1016/j.jesp.2006.12.006

[ref46] Netemeyer R. G. Johnston M. W. Burton S. (1990). Analysis of role conflict and role ambiguity in a structural equations framework. J. Appl. Psychol. 75, 148–157. doi: 10.1037/0021-9010.75.2.148

[ref47] Newman A. Schwarz G. Cooper B. Sendjaya S. (2017). How servant leadership influences organizational citizenship behavior: The roles of LMX. Empowerment and proactive personality. J. Bus. Ethics 145, 49–62. doi: 10.1007/s10551-015-2827-6

[ref48] Nieto M. J. Santamaria L. Fernandez Z. (2015). Understanding the innovation behavior of family firms. J. Small Bus. Manage. 53, 382–399. doi: 10.1111/jsbm.12075

[ref49] Okimoto T. G. Wenzel M. (2011). Third-party punishment and symbolic intragroup status. J. Expe. Soc. Psychol. 47, 709–718. doi: 10.1016/j.jesp.2011.02.001

[ref50] Oldham G. R. Hackman J. R. (2010). Not what it was and not what it will be: the future of job design research. J. Organ. Behav. 31, 463–479. doi: 10.1002/job.678

[ref51] Pan W. Sun L. Y. Chow I. H. S. (2012). Leader-member exchange and employee creativity: test of a multilevel moderated mediation model. J. Human Perform. 25, 432–451. doi: 10.1080/08959285.2012.721833

[ref52] Pearce J. L. Ramirez R. R. Branyiczki I. (2001). Leadership and the pursuit of status: effects of globalization and economic transformation. J. Adv. Global Lead. 2, 153–178. doi: 10.1016/S1535-1203(01)02118-9

[ref53] Phillips A. S. Bedeian A. G. (1994). Leader–follower exchange quality: the role of personal and interpersonal attributes. J. Acad. Manage. 37, 990–1001. doi: 10.2307/256608, PMID: 10137271

[ref54] Preacher K. J. Zyphur M. J. Zhang Z. (2010). A general multilevel SEM framework for assessing multilevel mediation. J. Psychol. Methods 15, 209–233. doi: 10.1037/a0020141 20822249

[ref55] Sarkawi M. N. Jaafar A. R. Shamsuddin J. Rahim N. (2016). Moderating effect of growth need strength on the relationship between job characteristics and job satisfaction. J. Int. Rev. Manage. Mark. 6, 212–216.

[ref56] Seibert S. E. Kraimer M. L. Crant J. M. (2001). What do proactive people do? A longitudinal model linking proactive personality and career success. J.Pers. Psychol. 54, 845–874. doi: 10.1111/j.1744-6570.2001.tb00234.x

[ref57] Shalley C. E. Gilson L. L. Blum T. C. (2009). Interactive effects of growth need strength, work context, and job complexity on self-reported creative performance. J. Acad. Manage. 52, 489–505. doi: 10.5465/amj.2009.41330806

[ref58] Sidanius J. Pratto F. Van Laar C. Levin S. (2004). Social dominance theory: its agenda and method. J. Polit. Psychol. 25, 45–880. doi: 10.1111/j.1467-9221.2004.00401.x

[ref59] Strubler D. C. Redekop B. W. (2010). Entrepreneurial human resource leadership: a conversation with Dwight Carlson. J. Human Res. Manage. 49, 793–804. doi: 10.1002/hrm.20368

[ref60] Tang Y. H. Mao J. H. (2020). The influence of individual perception of dissimilarity and workplace ostracism on knowledge sharing behavior: A test of two moderating effects. J. Sci. Res. Manage. 41, 200–208. doi: 10.19571/j.cnki.1000-2995.2020.04.021

[ref61] Volmer J. Spurk D. Niessen C. (2012). Leader-member exchange (LMX), job autonomy, and creative work involvement. J. Lead. Q. 23, 456–465. doi: 10.1016/j.leaqua.2011.10.005

[ref62] Wang Y. Liu J. Zhu Y. (2018). How does humble leadership promote follower creativity? The roles of psychological capital and growth need strength. J. Leadership Organ. Dev. 39, 507–521. doi: 10.1108/LODJ-03-2017-0069

[ref63] Wang B. Yang D. (2015). Anxious or differentiation? The impacting of the new generation of migrant workers’ growing demand strength on their organizational identification and intra-job embedded. J. Human Res. Sustain. Stud. 3, 82–91. doi: 10.4236/jhrss.2015.32012

[ref64] Wilson K. S. Sin H. P. Conlon D. E. (2010). What about the leader in leader-member exchange? The impact of resource exchanges and substitutability on the leader. Acad. Manag. Rev. 35, 358–372. doi: 10.5465/AMR.2010.51141654

[ref65] Yu H. Y. Kan L. W. Shang Y. J. (2020). Perceptions of cultural difference, communication mode and employee innovative behavior. J. Sci. Res. Manage. 41, 139–148. doi: 10.19571/j.cnki.1000-2995.2020.12.013

[ref66] Zhang Z. Wang M. Shi J. (2012). Leader-follower congruence in proactive personality and work outcomes: the mediating role of leader-member exchange. J. Acad. Manage. 55, 111–130. doi: 10.5465/amj.2009.0865

[ref67] Zhou J. George J. M. (2001). When job dissatisfaction leads to creativity: encouraging the expression of voice. J. Acad. Manage. 44, 682–696. doi: 10.2307/3069410

[ref68] Zhou Q. Hirst G. Shipton H. (2012). Promoting creativity at work: the role of problem-solving demand. J. Appl. Psychol. 61, 56–80. doi: 10.1111/j.1464-0597.2011.00455.x

